# Environmental factors influencing spotted hyena and lion population biomass across Africa

**DOI:** 10.1002/ece3.8359

**Published:** 2021-11-29

**Authors:** Angharad K. Jones, Simon P.E. Blockley, Danielle C. Schreve, Chris Carbone

**Affiliations:** ^1^ Department of Geography Royal Holloway University of London Egham UK; ^2^ Institute of Zoology Zoological Society of London London UK; ^3^ Creswell Heritage Trust Creswell Crags Museum and Heritage Centre Worksop UK

**Keywords:** Africa, competition, *Crocuta crocuta*, large carnivores, *Panthera leo*, population biomass

## Abstract

The spotted hyena (*Crocuta crocuta* Erxleben) and the lion (*Panthera leo* Linnaeus) are two of the most abundant and charismatic large mammalian carnivores in Africa and yet both are experiencing declining populations and significant pressures from environmental change. However, with few exceptions, most studies have focused on influences upon spotted hyena and lion populations within individual sites, rather than synthesizing data from multiple locations. This has impeded the identification of over‐arching trends behind the changing biomass of these large predators. Using partial least squares regression models, influences upon population biomass were therefore investigated, focusing upon prey biomass, temperature, precipitation, and vegetation cover. Additionally, as both species are in competition with one other for food, the influence of competition and evidence of environmental partitioning were assessed. Our results indicate that spotted hyena biomass is more strongly influenced by environmental conditions than lion, with larger hyena populations in areas with warmer winters, cooler summers, less drought, and more semi‐open vegetation cover. Competition was found to have a negligible influence upon spotted hyena and lion populations, and environmental partitioning is suggested, with spotted hyena population biomass greater in areas with more semi‐open vegetation cover. Moreover, spotted hyena is most heavily influenced by the availability of medium‐sized prey biomass, whereas lion is influenced more by large size prey biomass. Given the influences identified upon spotted hyena populations in particular, the results of this study could be used to highlight populations potentially at greatest risk of decline, such as in areas with warming summers and increasingly arid conditions.

## Graphical abstract

This paper investigates the environmental influences upon spotted hyena (*Crocuta crocuta*) and lion (*Panthera leo*) biomass across Africa. The results indicate that spotted hyena biomass is more strongly influenced by environmental conditions than lion, with larger hyena populations in areas with warmer winters, cooler summers, less drought, and more semi‐open vegetation cover. Competition was found to have a negligible influence upon spotted hyena and lion populations, and environmental partitioning is suggested, with spotted hyena population biomass greater in areas with more semi‐open vegetation cover with spotted hyena most heavily influenced by the availability of medium‐sized prey biomass and lion by large size prey biomass.

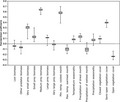

## 1 INTRODUCTION

The spotted hyena (*Crocuta crocuta* Erxleben) and the lion (*Panthera leo* Linnaeus) are two of the most widespread and abundant large mammalian carnivores in Africa (Hatton et al., 2015, and references therein). Nevertheless, despite their abundance, the lion's IUCN Red List classification is Vulnerable, with a decreasing population trend, and a population estimate in Africa of close to 20,000 individuals (Bauer et al., 2016). The spotted hyena fares better but its population is also decreasing, with an estimate of 27,000–47,000 individuals (Bohm & Höner, 2015). It is consequently critical to understand the factors influencing the population biomasses of these two species, to quantify better the limitations on their populations, aiding knowledge of drivers of population decline, and informing strategies for conservation. Given the current threat of climate change impacts, the focus of this paper will be on ecological factors, including the abundance of prey, the potential for competition, climate (temperature and precipitation variables), and vegetation cover.

Higher carnivore population density is primarily controlled by high prey biomass (Carbone & Gittleman, 2002) but in general, including the African savannah, predator biomass increases at a lower rate to prey biomass (Hatton et al., 2015). The densities of lion and spotted hyena are positively correlated with prey biomass (Périquet et al., 2015) and prey density (Cooper, 1989), respectively. Hayward et al. (2007) found that predator population densities were correlated with biomass of preferred prey and biomass of preferred prey weight range (i.e., biomass of prey weighing 190–550 kg in the case of lion; Hayward & Kerley, 2005). Van Orsdol et al. (1985) found that it was prey biomass during the season of lowest food availability that was most important food metric influencing lion population density.

Population size may also be influenced by competition (Carbone & Gittleman, 2002), with a reduction in preferred prey resulting in suboptimal foraging (Hayward & Kerley, 2008), predation, and disease (Kissui & Packer, 2004). Spotted hyena and lion compete with each other, and with other carnivores, for food through both interference competition (direct interactions, Amorós et al., 2020; Kruuk, 1972) and exploitation competition (the use of the same resource by different species, Amorós et al., 2020; Hayward & Kerley, 2008; Périquet et al., 2015). The success of interference competition depends on factors such as the numbers of spotted hyenas relative to the numbers of lions at a carcass, and whether or not an adult male lion is present (Cooper, 1991; Höner et al., 2002). It is therefore important to explore whether competition with other carnivores influences population biomass of spotted hyena and lion.

Competition may be reduced through temporal partitioning (carnivores being active at different times of the day, Hofer, 1998; Mills, 1998; Périquet et al., 2015; Schaller, 1972) or through spatial partitioning, such as different carnivore species occupying different types of vegetation (Schaller, 1972). Targeting of different prey age classes, in addition to frequency of scavenging, also separates carnivore species (Mills, 1990).

As well as prey biomass and competition, other factors may influence lion and spotted hyena populations. For example, Celesia et al. (2010) suggested that rainfall, temperature, and elevation were more important influences upon lion density. Ogutu and Dublin (2002) also found that rainfall influences lion density. The triangle area of the Maasai Mara National Reserve has lower lion density than elsewhere in the reserve. There, the low precipitation in the dry season has an indirect affect due to the resulting low food availability. In the wet season, the area becomes waterlogged, which results in greater disease prevalence (Ogutu & Dublin, 2002).

Precipitation is also important for spotted hyena populations. Although spotted hyenas may obtain much of their water requirements from fresh carcasses in arid areas, population density is higher in areas of reliable water sources (Cooper, 1989). Examples of arid areas with low population density of spotted hyena include the Kalahari Gemsbok National Park in South Africa (Mills, 1990) and the Tsauchab River Valley in Namibia (Fouche et al., 2020). In addition, Gasaway et al. (1991) suggested that arid conditions may reduce spotted hyena populations if prey is scarce and most of the food comes in the form of desiccated carcasses. Temperature may also be important for spotted hyenas, which are inactive during the hottest parts of the day (Hayward & Hayward, 2007). More specifically, Cooper (1990) found that spotted hyena individuals were unable to hunt in temperatures above about 20°C.

Given the aforementioned potential influences of biomass and climate upon spotted hyena and lion populations, in addition to the reduction of competition through various mechanisms of niche separation, any changes to prey biomass, temperature, precipitation, or vegetation openness due to climate change or human influence may be a concern for future populations. For example, Wolf and Ripple (2016) demonstrated that declining populations of prey species may increase the vulnerability of carnivore populations, although the lion and spotted hyena were not among the most vulnerable species. Similarly, Sandom et al. (2017) stated that declining prey is of particular threat to large felids (weighing more than 15 kg), primarily relying upon larger prey. As for changing climatic conditions, an example is the African wild dog (*Lycaon pictus*). There is concern that switching from diurnal to nocturnal activity in response to hot temperatures by the African wild dog may be insufficient to make up for lost daytime hunting during denning seasons, indicating potential negative impacts of future rise in temperature for this species (Rabaiotti & Woodroffe, 2019).

In this paper, the variation in population biomass density in the spotted hyena and the lion and their corresponding environmental correlates (other predator biomass, prey biomass, temperature, precipitation, and vegetation cover) is explored. With the notable exceptions of Cooper (1989), Hayward, O’Brien, and Kerley (2007), Celesia et al. (2010), and Périquet et al. (2015), few studies have attempted this type of synthetic analysis, leaving many aspects of the detailed interactions of spotted hyenas and lions to be explored. This paper therefore takes the novel approach of looking at the influences across spotted hyena and lion populations in Africa, and the breadth of this study means it can provide a much wider overview on factors influencing spotted hyena and lion population decline.

## 2 MATERIALS AND METHODS

### 2.1 Sites and data

**FIGURE 1:**
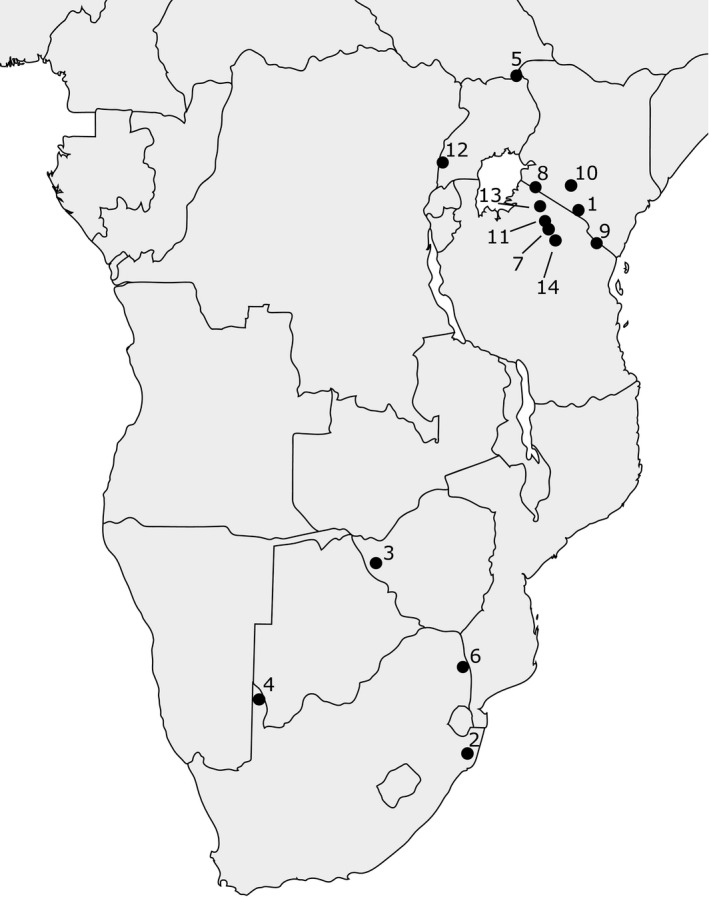
Location of sites used in the spotted hyena and lion biomass analyses. 1. Amboseli National Park, Kenya. 2. Hluhluwe iMfolozi National Park, South Africa. 3. Hwange National Park, Zimbabwe. 4. Kalahari Gemsbok National Park, South Africa. 5. Kidepo Valley National Park, Uganda. 6. Kruger National Park, South Africa. 7. Lake Manyara National Park, Tanzania. 8. Maasai Mara National Reserve, Kenya. 9. Mkomazi Game Reserve, Tanzania. 10. Nairobi National Park, Kenya. 11. Ngorongoro Crater, Tanzania. 12. Queen Elizabeth National Park, Uganda. 13. Serengeti ecosystem, Tanzania. 14. Tarangire National Park, Tanzania

The influences of environmental variables upon spotted hyena and lion biomass were investigated from 14 published sites across Africa (Figure 1). From these sites, data were obtained on predator and prey biomass, temperature metrics, precipitation metrics, and vegetation cover.

The predator and prey population biomass density data were obtained from a database in Hatton et al. (2015), who collated animal abundance data from the literature for locations across Africa. Sites were excluded from the present study if spotted hyena were absent, if the abundance of a species was uncertain, if spotted hyena abundance was combined with that of another hyaenid, or if the boundary of the site could not be determined. In total, 30 datasets were included in the biomass analyses from different years spanning 1962 to 2009 (Figure 1 and Table 1).

**TABLE 1:** Sites from Hatton et al.’s (2015) database included in the spotted hyena and lion biomass analyses

Site	Year (season)
Amboseli National Park, Kenya	2007
Hluhluwe iMfolozi National Park, South Africa	1982, 2000
Hwange National Park, Zimbabwe	1973
Kalahari Gemsbok National Park, South Africa	1979
Kidepo Valley National Park, Uganda	2009
Kruger National Park, South Africa	1975, 1984, 1997, 2009
Lake Manyara National Park, Tanzania	1970
Maasai Mara National Reserve, Kenya	1992, 2003
Mkomazi Game Reserve, Tanzania	1970 (dry), 1970 (wet)
Nairobi National Park, Kenya	1966, 1976, 2002
Ngorongoro Crater, Tanzania	1965, 1978, 1988, 1997, 2004
Queen Elizabeth National Park, Uganda	2009
Serengeti ecosystem, Tanzania	1971, 1977, 1986, 2003
Tarangire National Park, Tanzania	1962 (dry), 1962 (wet)

In addition to spotted hyena and lion biomass, the biomasses of other large predators were collected and combined for each site. Large predators are here regarded as those with an adult body mass of over 20 kg. In Africa, there are seven large mammalian predators: spotted hyena, brown hyena (*Parahyaena brunnea*), striped hyena (*Hyaena hyaena*), lion, leopard (*Panthera pardus*), cheetah (*Acinonyx jubatus*), and African wild dog. However, striped hyena was not included as data for this species are scarce. This is with the exception of the Tarangire National Park, Tanzania, where striped hyena abundance data were provided in lieu of brown hyena abundance (Hatton et al., 2015, and references therein). The striped hyena is solitary and occurs at low densities (Hofer & Mills, 1998), so its exclusion from the present study should not greatly influence the results.

Hatton et al.’s (2015) database includes biomasses of potential prey species over 5 kg in weight. Prey were split into five body size classes, following the distinctions of Périquet et al. (2015): very small (<20 kg), small (20–120 kg), medium (120–400 kg), large (400–600 kg), and very large (>600 kg).

Unless otherwise stated in the original publications or by Hatton et al. (2015), the boundaries of the sites were taken to be the entire area, that is, the entire national park, national reserve, game reserve, or district. The Serengeti ecosystem datasets in Hatton et al. (2015) were derived from a number of different publications; therefore, the boundaries of this site were taken from a map of the Serengeti ecosystem (Hopcraft, 2008).

The climate variables used were as follows: maximum temperature of the warmest month, minimum temperature of the coolest month, temperature seasonality (as standard deviation), precipitation of the wettest month, precipitation of the driest month, and precipitation seasonality (as the coefficient of variation). All data are from WorldClim (Hijmans et al., 2005) and were derived from interpolated records of climate data recorded between the years 1950–2000. The variables were taken from the bioclimatic dataset at a resolution of 2.5 min. Each temperature and precipitation value was taken from the center of each site. The center point of each site was the point where the median latitude and longitude intersected. Median latitude was calculated from the most northerly and southerly latitudes of each location. The same was performed for longitude. This was done using Image Landsat Google Earth Pro (2013).

The vegetation data are taken from the University of Maryland Global Land Cover Classification at 1‐km resolution (Hansen et al., 1998, 2000) and obtained by the Advanced Very High Resolution Radiometer satellites between the years 1981 and 1994. For each site, the type of vegetation in each pixel (each 1 km^2^) was recorded along two transects with widths of 1 km. The north‐south transect ran through the center point of the site, to the most northern and southern boundaries. The equivalent procedure was conducted for the east‐west transect. The counts for both transects were then combined.

Vegetation types were split into three categories: (1) open vegetation (grassland), (2) semi‐open vegetation (wooded grassland, open shrubland), and (3) closed vegetation (evergreen broadleaf forest, deciduous broadleaf forest, woodland, closed shrubland; see Table 2). The percentage cover of each classification was calculated from the transect counts. Some transects fell over pixels classed as water, cropland, or bare ground. These were excluded from the percentage calculations as it was assumed that spotted hyenas and lions would not be regularly inhabiting these areas.

**TABLE 2:** Vegetation classes and descriptions from the University of Maryland Global Land Cover Classification at 1‐km resolution (Hansen et al., 1998, 2000), and classes used in the present study

Vegetation class	Description	Vegetation class in present study
Evergreen broadleaf forest	Dominated by trees Tree canopy cover >60% Tree height >5 m Most trees remain green all year Canopy never without green foliage	Closed vegetation
Deciduous broadleaf forest	Dominated by trees Tree canopy cover >60% Tree height >5 m Trees shed their leaves simultaneously in response to dry or cold seasons	
Woodland	Herbaceous or woody understories Tree canopy cover >40% and <60% Tree height >5 m Trees evergreen or deciduous	
Closed shrubland	Dominated by shrubs Shrub canopy cover >40% Tree canopy cover <10% Shrub height <5 m Shrubs evergreen or deciduous Remaining area barren or herbaceous	
Wooded grassland	Herbaceous or woody understories Tree canopy cover >10% and <40% Tree height >5 m Trees evergreen or deciduous	Semi‐open vegetation
Open shrubland	Dominated by shrubs Shrub canopy cover >10% and <40% Shrub height <2 m Shrubs evergreen or deciduous Remaining area barren or annual herbaceous cover	
Grassland	Continuous herbaceous cover Tree or shrub canopy cover <10%	Open vegetation

Full details of the biomass, climate, and vegetation data for each site are included in the https://doi.org/10.5061/dryad.prr4xgxmj.

### 2.2 Statistical analyses

The relationships between key variables (prey biomass, predator biomass, temperature variables, precipitation variables, and vegetation cover) were analyzed initially to enable an appropriate statistical analyses strategy. In many cases where it is appropriate to test the responses of species population data to environmental influences, where there may be several dependent variables, multiple regression analyses is often used (see Carrascal et al., 2009). Spearman rank order correlations revealed significant correlations between many of the independent environmental variables of interest here (Table A1), meaning that multiple regression analysis could not be applied to determine the relationship between dependent and independent variables, since it cannot accommodate multicollinearity (Carrascal et al., 2009; Mac Nally, 1996, 2000). Similarly, the presence of 16 independent variables in this study also prevented the use of hierarchical partitioning (see Olea et al., 2010).

Partial least squares (PLS) regression was therefore chosen. In a comparison of three statistical tests (multiple regression, principal components analysis followed by multiple regression, and PLS), Carrascal et al. (2009) found that PLS performed better under multicollinearity, even with low sample sizes. PLS is also ideal in the current study given that there are 16 independent variables and 30 datasets: PLS is useful when “the number of predictor variables is similar to or higher than the number of observations” (Carrascal et al. (2009, p. 682).

Prior to the assessment of the influence of environmental conditions upon spotted hyena and lion biomass, the biomass, temperature, and precipitation datasets were base‐10 logarithmically transformed to reduce skew and to avoid autocorrelation. Some datasets contained values of zero that could not be log transformed. Where this was the case, the value of zero was converted to a value a unit of magnitude lower than the lowest nonzero value in the dataset. For example, if the lowest value was one, the zero was converted to 0.1, and then base‐10 logarithmically transformed.

The vegetation cover data are expressed as percentages and therefore could not simply be logarithmically transformed. Percentage data suffer from the Unit Sum auto‐correlation problem whereby the value of one variable is dependent on the value of the other variables that are used to calculate the percentage (Aitchison, 1982; Pollard et al., 2006). To avoid this, the vegetation data were transformed by the centered log‐ratio, following Kucera and Malmgren (1998) and Pollard et al. (2006):

$$g\left(x\right)={\left(x_{1}\ldots x_{d}\right)}^{1/d}$$

where *g* is the geometric mean of the vegetation category counts for each site, *x* is the count value of each vegetation category, and *d* is the number of vegetation categories. The ratio of a vegetation category count and the geometric mean was then calculated and base‐10 logarithmic transformed:

$$\text{clr(}x)=\log10\left(\frac{x}{g(x)}\right)$$

where clr is the centred log‐ratio, and log10 is the base‐10 logarithmic transformation.

In the present study, each PLS produced a *p*‐value and *r* ^2^ value. The strength of association of each independent variable with the dependent variable was indicated by the standardized coefficients.

The results were assessed for outliers and leverage points. A site was classed as an outlier if its standardized residual had a value greater or less than two. A site was deemed as a leverage point if its value fell beyond the vertical leverage reference line (LRL), which was calculated by:

$$\text{LRL}=\frac{2m}{n}$$

where *m* is the number of components in the PLS, and *n* is the number of observations (Minitab Inc., 2010).

To assess the effect of underlying variation in the data included in the PLS models, each model was re‐run 29 times, excluding one site each time. This indicated whether some sites were disproportionately influencing the results, and whether spotted hyena and lion biomasses varied consistently with environmental conditions across all sites. The standardized coefficients were displayed in boxplots to highlight the variables with consistently positive or negative values, which would indicate that there was a consistent relationship between the dependent and independent variable, regardless of which sites were included in the model.

Statistical analyses were performed using Minitab^®^ Statistical Software 17.3.1, Minitab^®^ Statistical Software 18.1 and PAST 3.12 (Hammer et al., 2001).

## 3 RESULTS

The *r* ^2^ and *p*‐values of the PLS regressions for both spotted hyena and lion are summarized in Table 3. PLS 1a assessed influences upon spotted hyena biomass and the whole model is significant in explaining spotted hyena biomass, with a *p*‐value of <.05 and an *r* ^2^ value of .84. Assessing the influences upon lion biomass, PLS 2a is also significant in explaining variation in lion biomass with a *p*‐value of <.05, yet it has a lower *r* ^2^ value of .61.

**TABLE 3:** Details of the partial least squares regressions run on spotted hyena and lion biomass

PLS regression	Dependent variable	*p*‐Value	*r* ^2^ Value
PLS 1a	Spotted hyena biomass	<.05	.837
PLS 1b	Spotted hyena biomass (without Kalahari)	<.05	.957
PLS 2a	Lion biomass	<.05	.608
PLS 2b	Lion biomass (without Kalahari)	<.05	.967

**FIGURE A1:**
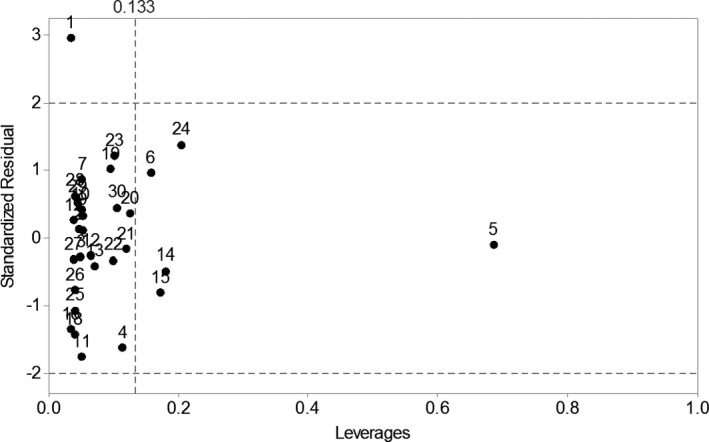
Standardized residuals against leverage values for each site in PLS 1a, with spotted hyena biomass as the dependent variable. The horizontal lines indicate the outlier boundaries. The vertical line represents the leverage reference line boundary. The numbers on the points correspond to sites as follows: 1. Amboseli National Park, 2007, 2. Hluhluwe iMfolozi National Park, 1982, 3. Hluhluwe iMfolozi National Park, 2000, 4. Hwange National Park, 1973, 5. Kalahari Gemsbok National Park, 1979, 6. Kidepo Valley National Park, 2009, 7. Kruger National Park, 1975, 8. Kruger National Park, 1984, 9. Kruger National Park, 1997, 10. Kruger National Park, 2009, 11. Lake Manyara National Park, 1970, 12. Maasai Mara National Reserve, 1992. 13. Maasai Mara National Reserve, 2003, 14. Mkomazi Game Reserve, 1970 (dry), 15. Mkomazi Game Reserve, 1970 (wet), 16. Nairobi National Park, 1966, 17. Nairobi National Park, 1976, 18. Nairobi National Park, 2002, 19. Ngorongoro Crater, 1965, 20. Ngorongoro Crater, 1978, 21. Ngorongoro Crater, 1988, 22. Ngorongoro Crater, 1997, 23. Ngorongoro Crater, 2004, 24. Queen Elizabeth National Park, 2009, 25. Serengeti ecosystem, 1971, 26. Serengeti ecosystem, 1977, 27. Serengeti ecosystem, 1986, 28. Serengeti ecosystem, 2003, 29. Tarangire National Park, 1962 (dry), 30. Tarangire National Park, 1962 (wet)

The plot of standardized residuals against leverages (Figure A1) was assessed for outliers and leverage points in PLS 1a. Only one site is an outlier: Amboseli National Park, Kenya. The leverage reference line (LRL) value was estimated at 0.13, and four sites fall just beyond this value. A fifth site, Kalahari Gemsbok National Park in South Africa, has an extreme leverage value of 0.69.

**FIGURE A2:**
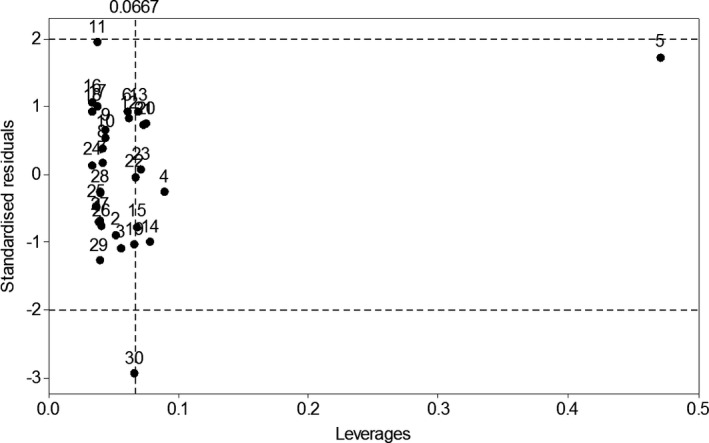
Standardized residuals against leverage values for each site in PLS 2a, with lion biomass as the dependent variable. The horizontal lines indicate the outlier boundaries. The vertical line represents the leverage reference line boundary. The numbers on the points correspond to sites as follows: 1. Amboseli National Park, 2007, 2. Hluhluwe iMfolozi National Park, 1982, 3. Hluhluwe iMfolozi National Park, 2000, 4. Hwange National Park, 1973, 5. Kalahari Gemsbok National Park, 1979, 6. Kidepo Valley National Park, 2009, 7. Kruger National Park, 1975, 8. Kruger National Park, 1984, 9. Kruger National Park, 1997, 10. Kruger National Park, 2009, 11. Lake Manyara National Park, 1970, 12. Maasai Mara National Reserve, 1992. 13. Maasai Mara National Reserve, 2003, 14. Mkomazi Game Reserve, 1970 (dry), 15. Mkomazi Game Reserve, 1970 (wet), 16. Nairobi National Park, 1966, 17. Nairobi National Park, 1976, 18. Nairobi National Park, 2002, 19. Ngorongoro Crater, 1965, 20. Ngorongoro Crater, 1978, 21. Ngorongoro Crater, 1988, 22. Ngorongoro Crater, 1997, 23. Ngorongoro Crater, 2004, 24. Queen Elizabeth National Park, 2009, 25. Serengeti ecosystem, 1971, 26. Serengeti ecosystem, 1977, 27. Serengeti ecosystem, 1986, 28. Serengeti ecosystem, 2003, 29. Tarangire National Park, 1962 (dry), 30. Tarangire National Park, 1962 (wet)

For PLS 2a, only one site shows as an outlier: Tarangire National Park, wet season (Figure A2). The LRL value is 0.07 and a number of sites fall beyond this line. Like PLS 1a, only Kalahari Gemsbok National Park has an extreme leverage value (0.47) in PLS 2a.

As leverage points may have a strong influence upon the coefficients, PLS 1a and PLS 2a were run again without Kalahari Gemsbok National Park.

**FIGURE A3:**
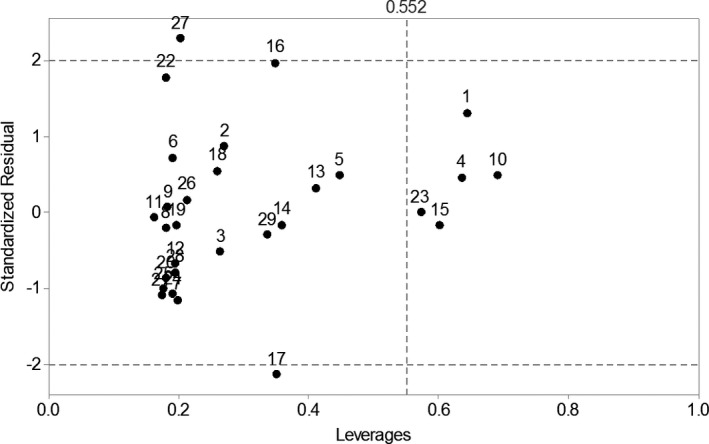
Standardized residuals against leverage values for each site in PLS 1b, with spotted hyena biomass as the dependent variable. The horizontal lines indicate the outlier boundaries. The vertical line represents the leverage reference line boundary. The numbers on the points correspond to sites as follows: 1. Amboseli National Park, 2007, 2. Hluhluwe iMfolozi National Park, 1982, 3. Hluhluwe iMfolozi National Park, 2000, 4. Hwange National Park, 1973, 5. Kidepo Valley National Park, 2009, 6. Kruger National Park, 1975, 7. Kruger National Park, 1984, 8. Kruger National Park, 1997, 9. Kruger National Park, 2009, 10. Lake Manyara National Park, 1970, 11. Maasai Mara National Reserve, 1992. 12. Maasai Mara National Reserve, 2003, 13. Mkomazi Game Reserve, 1970 (dry), 14. Mkomazi Game Reserve, 1970 (wet), 15. Nairobi National Park, 1966, 16. Nairobi National Park, 1976, 17. Nairobi National Park, 2002, 18. Ngorongoro Crater, 1965, 19. Ngorongoro Crater, 1978, 20. Ngorongoro Crater, 1988, 21. Ngorongoro Crater, 1997, 22. Ngorongoro Crater, 2004, 23. Queen Elizabeth National Park, 2009, 24. Serengeti ecosystem, 1971, 25. Serengeti ecosystem, 1977, 26. Serengeti ecosystem, 1986, 27. Serengeti ecosystem, 2003, 28. Tarangire National Park, 1962 (dry), 29. Tarangire National Park, 1962 (wet)

The new PLS, with spotted hyena biomass as the dependent variable and excluding the Kalahari (PLS 1b), is again significant with a *p*‐value of <.05 and has a greater *r* ^2^ value of .96. Analysis of the plot of standardized residuals versus leverages for PLS 1b (Figure A3) reveals that Nairobi National Park (Kenya from 2002) and the Serengeti ecosystem (Tanzania from 2003) are both outliers, although they do not fall far beyond the outlier reference line. Five sites are classed as leverage points: Amboseli National Park in Kenya, Hwange National Park in Zimbabwe, Lake Manyara National Park in Tanzania, Nairobi National Park in Kenya from 1966, and Queen Elizabeth National Park in Uganda. However, with leverage values ranging from 0.57 to 0.69, these sites are not far beyond the LRL value of 0.55, and so were retained in the analysis.

**FIGURE A4:**
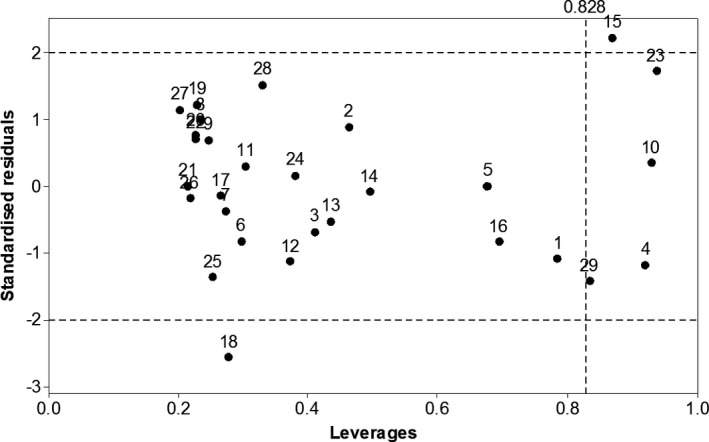
Standardized residuals against leverage values for each site in PLS 2b, with lion biomass as the dependent variable. The horizontal lines indicate the outlier boundaries. The vertical line represents the leverage reference line boundary. The numbers on the points correspond to sites as follows: 1. Amboseli National Park, 2007, 2. Hluhluwe iMfolozi National Park, 1982, 3. Hluhluwe iMfolozi National Park, 2000, 4. Hwange National Park, 1973, 5. Kidepo Valley National Park, 2009, 6. Kruger National Park, 1975, 7. Kruger National Park, 1984, 8. Kruger National Park, 1997, 9. Kruger National Park, 2009, 10. Lake Manyara National Park, 1970, 11. Maasai Mara National Reserve, 1992. 12. Maasai Mara National Reserve, 2003, 13. Mkomazi Game Reserve, 1970 (dry), 14. Mkomazi Game Reserve, 1970 (wet), 15. Nairobi National Park, 1966, 16. Nairobi National Park, 1976, 17. Nairobi National Park, 2002, 18. Ngorongoro Crater, 1965, 19. Ngorongoro Crater, 1978, 20. Ngorongoro Crater, 1988, 21. Ngorongoro Crater, 1997, 22. Ngorongoro Crater, 2004, 23. Queen Elizabeth National Park, 2009, 24. Serengeti ecosystem, 1971, 25. Serengeti ecosystem, 1977, 26. Serengeti ecosystem, 1986, 27. Serengeti ecosystem, 2003, 28. Tarangire National Park, 1962 (dry), 29. Tarangire National Park, 1962 (wet)

The PLS of lion biomass without Kalahari National Park (PLS 2b) is again significant with a *p*‐value of <.05. The *r* ^2^ value is greater at .97. Nairobi National Park (Kenya from 1966) and Ngorongoro Crater (Tanzania from 1965) were identified as outliers, although they do not fall far beyond the boundaries in the plot of standardized residuals versus leverages (Figure A4). Furthermore, five sites were identified as leverage points. However, with values ranging from 0.83 to 0.94, and relative to the LRL of 0.83, they are not extreme values and were retained in the model.

To assess the validity of the results, PLS 1b was re‐run 29 times, removing one site each time. All runs were significant with *p*‐values of <.05. The *r* ^2^ values ranged from .95 to .97 (Table A2), indicating that most of the variation in spotted hyena biomass was explained by each PLS run, regardless of the site that was removed. This justifies retaining the sites that fell just beyond the LRL; removal of them did not greatly alter the results. By contrast, the re‐runs of PLS 2b indicate that there was considerable variation in the results when some sites were removed. The *p*‐values are <.05 for each run, indicating that the regressions are significant. However, the *r* ^2^ values range from .98 to .56 (Table A3), indicating that there is much variation in lion biomass that is unexplained by the variables. The PLS regressions without the following sites have the lowest *r* ^2^ values: Kidepo Valley National Park, Lake Manyara National Park, Mkomazi Game Reserve (dry), Mkomazi Game Reserve (wet), Nairobi National Park (1976), Nairobi National Park (2002), Serengeti ecosystem (1971), and Serengeti ecosystem (2003). Of these, only Lake Manyara was originally identified as a leverage point for PLS 2b (Figure A4), again justifying inclusion of all the sites that fell just beyond the LRL.

For re‐runs of PLS 1b (spotted hyena as the dependent variable), the confidence intervals of the standardized coefficients are low, ranging from 0.01 for closed vegetation cover, to 0.02 for minimum temperature of the coolest month (Table 4). This indicates that confidence can be placed in the results, as no single site alters the results. Again, this is different for the re‐runs of PLS 2b (lion as the dependent variable), which are mostly larger than for PLS 1b. The confidence intervals ranged from 0.01 for very large prey biomass, to 0.19 for temperature of the warmest month (Table 5).

**TABLE 4:** Standardized coefficient means and confidence intervals (CI) for repeated runs of PLS 1b, with spotted hyena biomass as the dependent variable

Independent variable	Standardized coefficient mean	Standardized coefficient CI	Standardized coefficient minimum CI	Standardized coefficient maximum CI
Lion biomass	−0.035	0.011	−0.047	−0.024
Other predator biomass	−0.065	0.014	−0.079	−0.050
Total biomass very small prey	0.306	0.019	0.287	0.325
Total biomass small prey	0.136	0.018	0.118	0.154
Total biomass medium prey	0.635	0.011	0.624	0.647
Total biomass large prey	0.111	0.013	0.098	0.124
Total biomass very large prey	−0.012	0.011	−0.023	−0.001
Minimum temperature coolest month	0.577	0.021	0.555	0.598
Maximum temperature warmest month	−0.096	0.015	−0.111	−0.081
Temperature seasonality	0.082	0.009	0.074	0.091
Precipitation driest month	0.136	0.012	0.123	0.148
Precipitation wettest month	−0.102	0.019	−0.121	−0.084
Precipitation seasonality	0.073	0.016	0.058	0.089
Closed vegetation	0.094	0.008	0.087	0.102
Semi‐open vegetation	0.395	0.012	0.383	0.406
Open vegetation	−0.234	0.009	−0.243	−0.225

**TABLE 5:** Standardized coefficient means and confidence intervals (CI) for repeated runs of PLS 2b, with lion biomass as the dependent variable

Independent variable	Standardized coefficient mean	Standardized coefficient CI	Standardized coefficient minimum CI	Standardized coefficient maximum CI
Spotted hyena biomass	0.104	0.082	0.031	0.073
Other predator biomass	−0.005	0.155	0.059	−0.064
Total biomass very small prey	0.812	0.426	0.162	0.650
Total biomass small prey	0.187	0.098	0.037	0.150
Total biomass medium prey	0.230	0.142	0.054	0.176
Total biomass large prey	0.123	0.058	0.022	0.101
Total biomass very large prey	0.044	0.027	0.010	0.034
Minimum temperature coolest month	−0.474	0.306	0.116	−0.590
Maximum temperature warmest month	0.674	0.488	0.186	0.488
Temperature seasonality	−0.736	0.431	0.164	−0.900
Precipitation driest month	0.194	0.130	0.049	0.145
Precipitation wettest month	−0.398	0.327	0.124	−0.523
Precipitation seasonality	0.390	0.282	0.107	0.283
Closed vegetation	0.180	0.086	0.033	0.147
Semi‐open vegetation	−0.703	0.429	0.163	−0.866
Open vegetation	0.087	0.082	0.031	0.056

**FIGURE 2:**
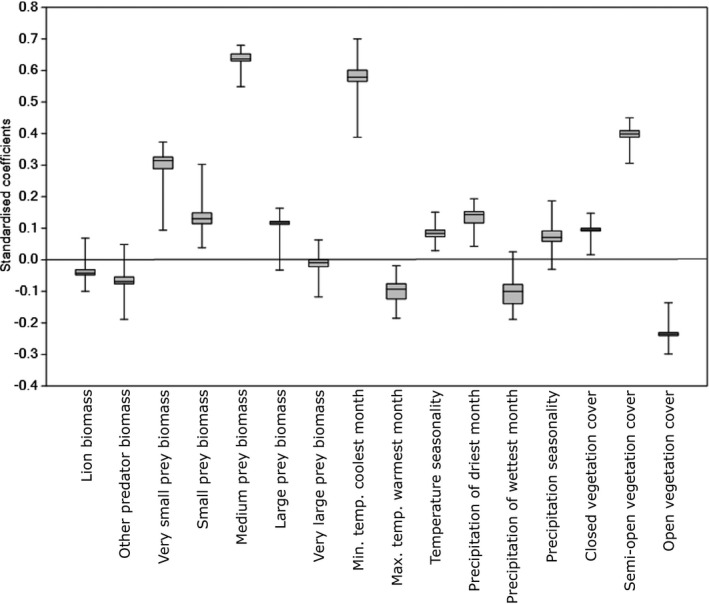
Standardized coefficients from repeated runs of PLS 1b, with spotted hyena biomass as the dependent variable

In the plot of the standardized coefficients for each run of PLS 1b (Figure 2), the coefficients that plot far from zero indicate importance in explaining spotted hyena biomass variation. The largest standardized coefficients are medium prey biomass and minimum temperature of the coolest month, both of which are positively associated with spotted hyena biomass. Other important variables include very small prey biomass and semi‐open vegetation on the positive side, and open vegetation on the negative side. Coefficients of other variables (lion and other predator biomasses, large and very large prey biomasses, precipitation seasonality) cluster around zero, suggesting that these hold little importance in explaining the variation in spotted hyena biomass.

**FIGURE 3:**
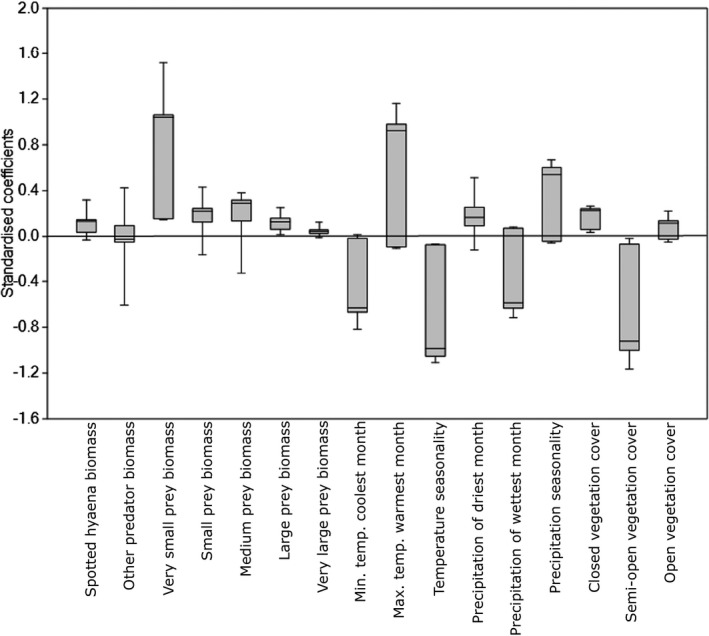
Standardized coefficients for repeated runs of PLS 2b, with lion biomass as the dependent variable

The graph of standardized coefficients from repeated runs of PLS 2b (Figure 3), assessing lion biomass, indicates that the removal of individual sites has a large influence on the PLS results. Most of the variables have coefficient values that are both positive and negative. Only two variables have coefficients that are consistently negative: temperature seasonality and semi‐open vegetation cover. Three variables have coefficients that are consistently positive: very small prey biomass, large prey biomass, and closed vegetation cover. Despite this, all these variables have coefficients from some runs that are close to zero. There is therefore no indication that any variables are consistently and strongly related to lion biomass, contrasting with the many more variables strongly related *to* spotted hyena biomass in PLS 1b.

## 4 DISCUSSION

Using PLS regression, the influences upon spotted hyena and lion biomass were assessed, focusing on competition, prey biomass, temperature, precipitation, and vegetation cover. The initial model runs (PLS 1a for spotted hyena biomass, and PLS 2a for lion biomass) indicated that the dataset from the Kalahari Gemsbok National Park (South Africa) likely had a strong influence on the results. The Kalahari Gemsbok National Park differs from other sites as it has the lowest abundance of spotted hyena with a biomass of 0.47 kg/km^2^. Additionally, Hatton et al. (2015) noted that the prey abundances recorded from the Kalahari were higher than previous estimates, so there were fewer predators than may have been expected given the prey biomass. This variation in prey abundance may be due to the correlation between prey and rainfall, the latter being unpredictable in the area (Mills, 1990). The extreme leverage value and the potential lag of predator abundance behind prey abundance mean that removing the site and re‐running the models (PLS 1b for spotted hyena biomass and PLS 2b for lion biomass) was an appropriate decision.

The repeated runs of PLS 1b provided further justification for excluding the Kalahari. Despite the removal of each site in turn, all runs reveal similar *r* ^2^ values to the original PLS 1b, and all *r* ^2^ values are higher than that of PLS 1a, indicating that more of the variation in spotted hyena biomass was explained by the models excluding the Kalahari. The similarity of all PLS 1b runs allows confidence to be placed in the assumption that the results are representative of spotted hyena populations.

Biomass of medium‐sized prey has the strongest overall influence on spotted hyena biomass. Despite spotted hyenas being adaptable in the prey that they target (Hayward, 2006; Mills, 1990), this result is corroborated by known preferred prey weights of 56–182 kg (Hayward & Kerley, 2008), equivalent to small‐ to medium‐sized prey in this study.

Similarly, the positive association between lion biomass and large‐sized prey biomass may be explained by the fact that lions most commonly target prey weighing 190–550 kg (Hayward & Kerley, 2005), equivalent to medium‐ to large‐sized prey species in this study. Further, large prey provide more energy intake for large predators, which is necessary to offset energy expended, including that expended while hunting (which is particularly high for predators of large body mass such as lion, Carbone et al., 2007).

The positive association with very small‐size prey biomass revealed here was unexpected for lion, as indeed it was for spotted hyena. Very small prey (weighing <20 kg, including Thomson's gazelle (*Eudorcas thomsonii*) and duiker species (*Cephalophus* spp.)) can provide an important food source, especially when larger prey are temporarily in short supply. This is the case in the Serengeti where resident Thomson's gazelle is the most abundant ungulate, and the most commonly targeted species by spotted hyena prior to the arrival of migrating blue wildebeest (*Connochaetes taurinus*; Cooper et al., 1999). In the Seronera area of the Serengeti, although lion predate small‐ to large‐sized prey species, during periods when these species are unavailable, lion will survive on very small‐sized prey, namely Thomson's gazelle (Schaller, 1972). The great significance of very small‐sized prey species may also reflect their importance in allowing the survival of lion when preferred (larger) prey are unavailable. A further example is seen in the importance of warthog (*Phacochoerus africanus*, here classed as small‐sized prey), in the diet of lions (Hayward et al., 2007). Further research is required to better understand within‐species carnivore abundance patterns in relation to the size and abundance of their prey base (following Carbone et al., 2011; Hatton et al., 2015).

Reliance of very small‐size prey may have implications for interspecies competition, particularly lion's and spotted hyena's competition with other carnivores that preferentially target smaller prey (e.g., African wild dog, Hayward & Kerley, 2008). This was observed in the Kafue National Park in Zambia: As larger sized prey populations decreased, smaller sized prey became more important in predator diets, meaning that there was more overlap in the diets of different predators (Creel et al., 2018). This evidence may therefore be used to highlight at‐risk populations (not only of spotted hyena and lion, but other predators, too) due to increased competition from prey structure changes that have necessitated reliance upon smaller prey.

The relationship between prey biomass and spotted hyena and lion biomasses agrees with Hatton et al. (2015) in that predator density and biomass are positively correlated. It also agrees with other studies, such as Cooper’s (1989) observation that higher spotted hyena densities occur in areas with large biomasses of resident prey populations, and Celesia et al.’s (2010) findings that lion density is positively influenced by herbivore biomass. In addition, Hayward, O’Brien, and Kerley (2007) found that preferred prey species biomass and preferred prey weight range biomass correlated with the densities of spotted hyena and lion and other African predators, and Van Orsdol et al. (1985) observed that lion density was influenced by prey biomass during the lean season (the season with lowest prey availability), although density was not influenced by prey biomass during the season with greatest prey availability. More broadly, prey populations have been observed to positively influence populations of other predators, such as wolf (*Canis lupus*) density and prey biomass in North America (Fuller & Murray, 1998), and tiger (*Panthera tigris*) density and prey density in India (Karanth et al., 2004). In addition to prey biomass, PLS 1b and 2b suggest that there are other hitherto undocumented influences upon spotted hyena and lion abundance, explored below.

The minimum temperature of the coolest month has a strong positive relationship with spotted hyena biomass, suggesting that spotted hyena is averse to the very coldest temperatures, that is, spotted hyena populations are greater when winter temperatures are warmer. Although Cooper (1990) and Hayward and Hayward (2007) indicated that spotted hyenas are inactive during the warmest part of the day, this result does not conflict with those studies, as the coolest month temperatures in this study range from 5.4 to 16.8°C (Hijmans et al., 2005). These temperatures are lower than the maximum tolerated temperature for hunting of 20°C (Cooper, 1990).

The maximum temperature of the warmest month has a negative relationship with spotted hyena biomass, although its potential influence is lower than for winter temperatures. This is supported by Cooper (1990) who found that spotted hyena individuals were unable to hunt in temperatures above around 20°C. Indeed, the summer temperatures of sites included in the present study are all above 20°C, ranging from 25.1 to 33.7°C (Hijmans et al., 2005), although spotted hyena may circumvent this to an extent through crepuscular or nocturnal activities (Cooper, 1990; Hayward & Hayward, 2007). As spotted hyenas were able to hunt successfully on moonlit nights, and during the day when temperatures were cooler, Cooper (1990) concluded that it is temperature, rather than a need for darkness, that prompts this switch to nocturnal hunting. Very hot temperatures also lead to more rapid decomposition of carrion, thus limiting the period during which carcasses are available as a food source (DeVault et al., 2003). However, avoidance of high temperatures through nocturnal activity may be the reason why high temperatures have only a small influence on spotted hyena biomass.

The negative association between lion biomass and temperature seasonality suggests that lion abundance is greatest in areas that have either predominantly year‐round high temperatures, or predominantly year‐round low temperatures, but not great seasonal temperature fluctuations. The other temperature variables (minimum temperature of the coolest month and maximum temperature of the warmest month) do not appear to influence lion biomass, although Celesia et al. (2010) found that a different temperature variable (mean annual temperature) is positively correlated with lion density.

Precipitation has some influence upon the spotted hyena, notably adverse effects caused by very dry conditions such as lack of available water bodies. In addition, hot and dry conditions may lead to more rapid desiccation of carcasses, which are themselves important sources of water for spotted hyena, especially in periods of drought (Cooper, 1990; Cooper et al., 1999). This ability to source water from carcasses may be one of the reasons for the limited influence of precipitation.

None of the precipitation variables appear to influence lion biomass, in contrast to Celesia et al.’s (2010) finding that lion density is positively correlated with mean annual rainfall. However, this variable is different to the ones used in the present study (precipitation of the wettest month, precipitation of the driest month, and precipitation seasonality).

Perhaps unexpectedly, open vegetation cover was found to have a strong negative relationship with spotted hyena abundance, while semi‐open vegetation cover has a positive relationship. The *s*potted hyena often hunts by pursuing its prey (Kruuk, 1972; Mills, 1990), so it would seem logical that open conditions should be optimal but this is not the case. Moreover, there appears to be no consistent vegetation preference for den location, with dens having been observed in open grassland (Amboseli Airstrip, Kenya), plains rather than wooded areas (Serengeti and Ngorongoro Crater, Tanzania), savannah and forest patches (Comoé National Park, Côte d’Ivoire), and patches of shrub and isolated trees (Namibia‐Naukluft Park, Namibia, Faith, 2007; Henschel et al., 1979; Korb, 2000; Kruuk, 1972; Tilson et al., 1980). The negative effect of open vegetation is therefore difficult to explain.

The positive influence of semi‐open vegetation with spotted hyena biomass may be explained by it being an ideal area for hunting; Mills (1990) observed *C*. *crocuta* chasing its prey in areas of open shrubland or open woodland in the Kalahari, which is similar to the semi‐open vegetation category in the present study (open shrubland and wooded grassland; Table 2).

In contrast to the spotted hyena, semi‐open vegetation cover is negatively associated with lion biomass. Indeed, even in individual sites, spatial partitioning has been observed between spotted hyenas and lions. For example, in the Serengeti, spotted hyenas occupy the plains and woodland borders while lions occupy the plains, but are most frequently within wooded grassland (Schaller, 1972). However, this in itself presents a problem as wooded grassland is classed as semi‐open vegetation in the present study. Additionally, Périquet et al. (2015) suggested that some vegetation cover is needed to allow lions to ambush their prey.

An alternative explanation for the relationship with vegetation cover may lie in the limitations of the dataset. The data were collected between the years 1981 and 1994 (Hansen et al., 1998, 2000), and so any change in vegetation before or after this time period was not recorded. This dataset is nevertheless preferable to obtaining vegetation data from a multitude of sources, as at least the data classification is consistent between sites (Hansen et al., 1998, 2000). Small‐scale differentiation (below the 1‐km resolution of the dataset, Hansen et al., 1998, 2000) at individual sites may be another explanation.

The final point to consider is the influence of other predators, particularly through competitive interactions. Both lion and the other predators (brown hyena, cheetah, leopard, African wild dog) have no consistent positive or negative influences on spotted hyena abundance. This is similar for lion: spotted hyena and other predators do not appear to influence lion biomass. Although spotted hyenas and lions are frequently successful in obtaining food from other predators, the reverse can be true, with the success of direct interactions depending upon the persistence of the challenger, the number of individuals present, and the presence of males in the case of lions (Cooper et al., 1999; Höner et al., 2002; Kruuk, 1972; Mills, 1990; Schaller, 1972). Therefore, any negative influence of other predators may be largely cancelled out by spotted hyena and lion succeeding in other competitive interactions. Additionally, as suggested by the aforementioned findings from PLS 1b and 2b, and those of other studies, environmental partitioning (including vegetation, prey preference, and time of activity) may limit the negative impact of other predators upon spotted hyena and lion abundance (Hofer, 1998; Mills, 1990, 1998; Périquet et al., 2015; Schaller, 1972). However, lack of influence of lions upon spotted hyenas is contrary to studies that suggest low lion populations likely lead to greater or more stable spotted hyena populations. For example, Green et al. (2019) observed that in the Talek West area of the Maasai Mara National Reserve, spotted hyena populations increased, driven by greater spotted hyena cub and juvenile survival, which may have been due to a reduction in lion populations in the area. Further, M’soka et al. (2016) observed that spotted hyenas had high survival rates and stable population density in Liuwa Plan National Park in Zambia, interpreted in part due to the low population of lions.

Although the five variables discussed above are the only ones that have a consistent positive or negative association with lion biomass, many of the coefficients of these variables are close to zero, depending upon the site removed from the PLS 2b re‐runs. The overall lack of consistency between runs suggests that the conditions influencing lion biomass are site‐specific or that there are additional influences that were not considered in the analyses. This is backed up by the low *r* ^2^ values on some of the PLS runs, which suggest that a large proportion of the variation in lion biomass is not explained by the model. This is in contrast to PLS 1b, which consistently has high *r* ^2^ values, associated with spotted hyena biomass.

One potential influence that was not included in the models is disease, which may influence population sizes and may be a factor in PLS 1b, given that there are datasets from Ngorongoro Crater, from five different years. This has been observed in the Ngorongoro Crater, where an outbreak of stable flies (*Stomoxys calcitrans*) in 1962 (Fosbrooke, 1963, cited in Kissui & Packer, 2004), unknown diseases in 1994 and 1997, and a tick‐borne disease and the canine distemper virus (Kissui & Packer, 2004) have all impacted the lion population. The same area has also witnessed short‐term declines in spotted hyena population density through an outbreak of *Streptococcus equi ruminatorum* in 2002–3 (Höner et al., 2006). This resulted in an increased mortality rate and associated population decline. The disease also became more prevalent with greater interspecific competition and lower prey density, indicating the importance of food availability in influencing the impact of disease (Höner et al., 2012).

A further potential influence is humans. However, it is difficult to quantify human impact in a way that can be included in the model. Woodroffe (2000) assessed impacts of humans by including densities of people in states, districts, and counties in the study. However, this approach is not suitable in the present study given that the sites are not restricted by political boundaries, but are instead conservation areas. Further, as all sites included in this study have some type of protected status (e.g., national park, conservation area, game reserve; Table 1), this may also influence biomass: Variation in management of protected areas has been found to influence lion population sizes across Africa (Lindsey et al., 2017).

Finally, climate change may have influenced the results. As most sites have only one or two datasets, temporal change in precipitation and vegetation, and associated changes in prey and predator abundances would not have been picked up in the model. It is difficult to say which species would be most affected by this. Given that environmental variables closely relate to spotted hyena biomass, this species may be influenced by climatic changes, although perhaps the changes thus far have not been strong enough to influence the model (suggested through the high *r* ^2^ values of the model re‐runs, despite the overall lack of temporal environmental data). By contrast, the varying *r* ^2^ values in the lion model may suggest that this species has been strongly impacted by climatic change, and the lack of inclusion of temporal environmental data in the model may explain why the model was unable to strongly explain influences upon lion biomass.

It is acknowledged that this study has some, unfortunately, unavoidable limitations related to the dataset. For example, the biomass data for some sites is decades old. This may be problematic for two reasons. First, species biomass or, as mentioned, vegetation cover may have changed since the population studies were conducted at some sites. Second, the older methods used to determine population sizes may have been superseded by more accurate methods, such as the spatial capture–recapture used for determining lion density in Kenya (Elliot & Gopalaswamy, 2016, and further discussed by Braczkowski et al., 2020). This may be of particular concern for species of low population size. Indeed, Mills (1990) acknowledged that nocturnal species in particular, such as the brown hyena, are difficult to count and therefore difficult to determine density and biomass. Ideally, the study would be conducted with modern population estimates of predators and prey from all sites.

Further enhancement would be for the study to be repeated with additional sites, particularly targeting those in western Africa to assess whether the predator populations maintain the same relationship with environmental variables in a wider geographical area. Given that the Kalahari Gemsbok National Park was removed, there were also only three sites located in southern Africa (although this did account for seven datasets given observations from multiple years at two of these sites). However, the study did analyze the information that is currently available from numerous sites in eastern and southern Africa. Finally, the information gained here, particularly regarding the influences upon spotted hyena biomass could be used to inform conservation efforts. For example, populations that are potentially vulnerable to population decline might be identified, such as in areas with warmer summers and drier conditions, as the model indicates that spotted hyena biomass is negatively influenced by these variables.

In summary, the results indicate that spotted hyena is the more sensitive of the two species to environmental conditions in terms of impacts on biomass. This is surprising given the plasticity of spotted hyena's behavior, such as switching from crepuscular to nocturnal activity, and changing the vegetation that individuals occupied in response to increased human presence in the Talek region of the Maasai Mara National Reserve (Boydston et al., 2003).

The results of the study have potential implications for conservation, particularly of spotted hyena. Any increasing aridity and warmer summer temperatures with climate change are of concern, especially as spotted hyena are inactive during the hottest parts of the day (Cooper, 1990; Hayward & Hayward, 2007); increasing summer temperature may limit the time during which they can hunt and thus limit food intake. Further, changes in vegetation, especially a decrease of semi‐open vegetation through land management should also be considered as a potential negative threat to spotted hyena populations. Changes in the extent of closed vegetation may also be a factor influencing lion population biomass.

## CONFLICT OF INTEREST

The authors declare no conflict of interest.

## AUTHOR CONTRIBUTION

**Angharad K. Jones:** Conceptualization (equal); Data curation (lead); Formal analysis (equal); Investigation (lead); Methodology (equal); Writing‐original draft (lead); Writing‐review & editing (equal). **Simon P.E. Blockley:** Formal analysis (equal); Methodology (supporting); Writing‐review & editing (equal). **Danielle C. Schreve:** Conceptualization (equal); Formal analysis (equal); Methodology (equal); Supervision (lead); Writing‐review & editing (equal). **Chris Carbone:** Conceptualization (lead); Formal analysis (equal); Methodology (equal); Supervision (lead); Writing‐review & editing (equal).

## Supporting information

Supporting Information

## Data Availability

Location, biomass, climate, and vegetation cover data: Dryad https://doi.org/10.5061/dryad.prr4xgxmj

## APPENDIX 1

**TABLE A1:** Spearman rank order correlations between variables included in PLS 1a and 2a, with spotted hyena and lion biomass as the dependent variable

Spearman rank order statistic, p‐value	Other predator biomass	Very small prey biomass	Small prey biomass	Medium prey biomass	Large prey biomass	Very large prey biomass	Min. temperature of coldest month	Max. temperature of warmest month	Temperature seasonality	Rainfall of the driest month	Rainfall of the wettest month	Rainfall seasonality	Closed vegetation cover	Semi‐open vegetation cover	Open vegetation cover
Spotted hyena biomass	0.663 <.05	0.768 <.05	0.604 <.05	0.815 <.05	0.595 .001	0.122 .519	−0.121 .522	−0.546 .002	−0.259 .168	0.19 .314	0.498 .005	−0.062 .743	0.431 .017	−0.119 .531	−0.379 .039
Lion biomass	0.712 <.05	0.833 <.05	0.551 .002	0.64 <.05	0.615 <.05	0.057 .763	0.125 .509	−0.536 .002	−0.507 .004	0.299 .108	0.576 .001	−0.149 .431	0.388 .034	−0.418 .022	−0.248 .187
Other predator biomass		0.81 <.05	0.648 <.05	0.709 <.05	0.447 .013	0.055 .774	−0.219 .246	−0.474 .008	−0.069 .717	0.186 .326	0.409 .025	0.002 .99	0.416 .022	−0.203 .282	−0.336 .07
Very small prey biomass			0.608 <.05	0.803 <.05	0.581 .001	−0.045 .812	−0.009 .964	−0.695 <.05	−0.452 .012	0.219 .245	0.715 <.05	−0.044 .818	0.349 .059	−0.26 .166	−0.272 .146
Small prey biomass				0.498 .005	0.499 .005	0.186 .325	−0.106 .579	−0.552 .002	−0.448 .013	0.675 <.05	0.174 .359	−0.541 .002	0.267 .154	−0.09 .636	−0.081 .67
Medium prey biomass					0.341 .065	−0.14 .461	−0.103 .589	−0.607 <.05	−0.25 .182	0.14 .459	0.598 <.05	0.065 .732	0.204 .28	−0.133 .483	−0.189 .317
Large prey biomass						0.374 .042	0.088 .644	−0.384 .036	−0.372 .043	0.274 .143	0.315 .09	−0.217 .25	0.539 .002	−0.36 .051	−0.394 .031
Very large prey biomass							0.148 .434	0.338 .068	0.117 .537	0.154 .416	−0.245 .191	−0.228 .226	0.356 .054	−0.042 .826	−0.16 .397
Minimum temperature of coldest month								−0.017 .93	−0.396 .03	0.201 .288	0.352 .057	−0.237 .207	−0.177 .351	−0.671 <.05	0.513 .004
Maximum temperature of warmest month									0.698 <.05	−0.425 .019	−0.64 <.05	0.221 .24	−0.056 .767	0.314 .091	−0.072 .704
Temperature seasonality										−0.601 <.05	−0.452 .012	0.53 .003	0.088 .642	0.362 .049	−0.275 .141
Rainfall of the driest month											−0.147 .439	−0.957 <.05	−0.094 .621	−0.292 .117	0.408 .025
Rainfall of the wettest month												0.314 .091	0.241 .199	−0.421 .021	−0.13 .495
Rainfall seasonality													0.139 .462	0.238 .204	−0.434 .017
Closed vegetation cover														−0.315 .09	−0.835 <.05
Semi‐open vegetation cover															−0.09 .637
Open vegetation cover															

Note

Top value is the *r_s_* statistic. Bottom value is the *p*‐value. Yellow shaded boxes show correlations significant at 95% confidence.

**TABLE A2:** *r* ^2^ Values and *p*‐values of repeated runs of PLS 1b, with spotted hyena biomass as the dependent variable

Run no.	Removed site	*r* ^2^ Value	*p*‐Value
1	Amboseli National Park, Kenya, 2007	.961	<.05
2	Hluhluwe iMfolozi National Park, South Africa, 1982	.96	<.05
3	Hluhluwe iMfolozi National Park, South Africa, 2000	.958	<.05
4	Hwange National Park, Zimbabwe, 1973	.951	<.05
5	Kidepo Valley National Park, Uganda, 2009	.961	<.05
6	Kruger National Park, South Africa, 1975	.958	<.05
7	Kruger National Park, South Africa, 1984	.96	<.05
8	Kruger National Park, South Africa, 1997	.957	<.05
9	Kruger National Park, South Africa, 2009	.957	<.05
10	Lake Manyara National Park, Tanzania, 1970	.955	<.05
11	Maasai Mara National Reserve, Kenya, 1992	.956	<.05
12	Maasai Mara National Reserve, Kenya, 2003	.956	<.05
13	Mkomazi Game Reserve, Tanzania, 1970 (dry)	.95	<.05
14	Mkomazi Game Reserve, Tanzania, 1970 (wet)	.95	<.05
15	Nairobi National Park, Kenya, 1966	.959	<.05
16	Nairobi National Park, Kenya, 1976	.968	<.05
17	Nairobi National Park, Kenya, 2002	.967	<.05
18	Ngorongoro Crater, Tanzania, 1965	.954	<.05
19	Ngorongoro Crater, Tanzania, 1978	.953	<.05
20	Ngorongoro Crater, Tanzania, 1988	.956	<.05
21	Ngorongoro Crater, Tanzania, 1997	.959	<.05
22	Ngorongoro Crater, Tanzania, 2004	.958	<.05
23	Queen Elizabeth National Park, Uganda, 2009	.957	<.05
24	Serengeti ecosystem, Tanzania, 1971	.959	<.05
25	Serengeti ecosystem, Tanzania, 1977	.96	<.05
26	Serengeti ecosystem, Tanzania, 1986	.957	<.05
27	Serengeti ecosystem, Tanzania, 2003	.969	<.05
28	Tarangire National Park, Tanzania, 1962 (dry)	.96	<.05
29	Tarangire National Park, Tanzania, 1962 (wet)	.954	<.05

Note

Each run removed one site at a time.

**TABLE A3:** *r* ^2^ Values and *p*‐values of repeated runs of PLS 2b, with lion biomass as the dependent variable

Run no.	Removed site	*r* ^2^ Value	*p*‐Value
1	Amboseli National Park, Kenya, 2007	.971	<.05
2	Hluhluwe iMfolozi National Park, South Africa, 1982	.969	<.05
3	Hluhluwe iMfolozi National Park, South Africa, 2000	.968	<.05
4	Hwange National Park, Zimbabwe, 1973	.969	<.05
5	Kidepo Valley National Park, Uganda, 2009	.631	<.05
6	Kruger National Park, South Africa, 1975	.969	<.05
7	Kruger National Park, South Africa, 1984	.968	<.05
8	Kruger National Park, South Africa, 1997	.967	<.05
9	Kruger National Park, South Africa, 2009	.969	<.05
10	Lake Manyara National Park, Tanzania, 1970	.639	<.05
11	Maasai Mara National Reserve, Kenya, 1992	.966	<.05
12	Maasai Mara National Reserve, Kenya, 2003	.968	<.05
13	Mkomazi Game Reserve, Tanzania, 1970 (dry)	.555	<.05
14	Mkomazi Game Reserve, Tanzania, 1970 (wet)	.574	<.05
15	Nairobi National Park, Kenya, 1966	.979	<.05
16	Nairobi National Park, Kenya, 1976	.605	<.05
17	Nairobi National Park, Kenya, 2002	.608	<.05
18	Ngorongoro Crater, Tanzania, 1965	.983	<.05
19	Ngorongoro Crater, Tanzania, 1978	.967	<.05
20	Ngorongoro Crater, Tanzania, 1988	.964	<.05
21	Ngorongoro Crater, Tanzania, 1997	.967	<.05
22	Ngorongoro Crater, Tanzania, 2004	.966	<.05
23	Queen Elizabeth National Park, Uganda, 2009	.977	<.05
24	Serengeti ecosystem, Tanzania, 1971	.596	<.05
25	Serengeti ecosystem, Tanzania, 1977	.972	<.05
26	Serengeti ecosystem, Tanzania, 1986	.965	<.05
27	Serengeti ecosystem, Tanzania, 2003	.595	<.05
28	Tarangire National Park, Tanzania, 1962 (dry)	.972	<.05
29	Tarangire National Park, Tanzania, 1962 (wet)	.965	<.05

Note

Each run removed one site at a time.
